# Association between maternal blood lipids and neonatal hypoglycaemia in pregnancy with gestational diabetes mellitus: a cohort study

**DOI:** 10.1186/s12944-024-02168-z

**Published:** 2024-06-07

**Authors:** Mo Zhang, Xiaoqing Huang, Suiwen Lin, Bin Liu

**Affiliations:** 1 Department of Obstetrics and Gynaecology, The First Affiliated Hospital of Sun Yat-sen University, Guangzhou, 510080 China; 2 Guangdong Provincial Clinical Research Center for Obstetrical and Gynaecological Diseases, Guangzhou, China

**Keywords:** Neonatal hypoglycaemia, Gestational diabetes mellitus, Blood lipids, Prediction model, Nomogram

## Abstract

**Background:**

Gestational diabetes mellitus (GDM) prevalence is on the rise globally. Offspring of diabetic mothers face increased risk of neonatal hypoglycaemia (NH), and women with GDM have abnormal lipid profiles. However, there is no consensus on the link between maternal blood lipids and NH in infants from mothers with GDM. This study aimed to explore how maternal blood lipids affect NH.

**Methods:**

A retrospective cohort study was conducted at the First Affiliated Hospital of Sun Yat-sen University. Information on participants’ baseline characteristics and maternal metabolic profiles of glucose and lipids was collected. Significant variables from the univariate analysis were included in logistic regression, which was used to construct the predictive model for NH. A nomogram was constructed for visualizing the model and assessed using the area under the receiver operating characteristic (ROC) curve (AUC).

**Results:**

Neonatal capillary blood glucose (CBG) decreased rapidly in the first hour after birth, increased gradually from the first to the second hour, and then remained stable. In the NH group, 86.11% (502/583) of hypoglycaemia cases occurred within the first two hours after birth. Multivariate logistic regression suggested that the lipid indices of maternal apoprotein B/apoprotein A1 (Apo-B/Apo-A1) (odds ratio (OR) = 1.36, 95% confidence intervals (CIs): 1.049–1.764, *P* = 0.02) and apoprotein E (Apo-E) (OR = 1.014, 95% CIs: 1.004–1.024, *P* = 0.004) were positively associated with NH in neonates from mothers with GDM. Triglycerides (TGs) (OR = 0.883, 95% CIs: 0.788–0.986, *P* = 0.028) were inversely associated with NH. Maternal glycated haemoglobin (HbA1c), age, twin pregnancy and caesarean delivery also had predictive value of NH. The AUC of the nomogram derived from these factors for the prediction model of NH was 0.657 (95% CIs: 0.630–0.684).

**Conclusions:**

The present study revealed that the Apo-B/Apo-A1 and Apo-E levels were associated with an increased risk of NH. A nomogram was developed to forecast the risk of NH in babies born to mothers with GDM, incorporating maternal blood lipids, HbA1c, age, twin pregnancy, and caesarean section. The trajectory of glycaemia for neonates indicates the need for intensive CBG monitoring within 2 h of birth for neonates from mothers with GDM.

## Background

Gestational diabetes mellitus (GDM) is diabetes identified during middle or late pregnancy [1]. GDM is a prevalent pregnancy complication, and the prevalence of GDM has increased in many countries over time [2–4]. GDM leads to adverse outcomes for mothers and foetuses. For mothers, GDM raises the chances of caesarean section and hypertensive disorders of pregnancy (HDP) [5]. Women with prior GDM have an increased likelihood of developing type 2 diabetes post-pregnancy [6]. The offspring of diabetic women are at increased risk of multiple short- and long-term complications [7–9], such as macrosomia and neonatal hypoglycaemia (NH) [5]. Among these complications, NH is frequent and is associated with cerebral damage [10, 11]. Therefore, more attention should be given to NH in clinical practice to avoid neonatal adverse outcomes.

The identification of the predictors and onset time of NH may contribute to individualized assessment and the update of management strategies for high-risk newborns [12]. According to previous studies and clinical guidelines, maternal blood glucose abnormalities, small for gestational age (SGA) or large for gestational age (LGA) raises NH risk [11, 13], but other factors, such as maternal glycated haemoglobin (HbA1c), caesarean section and twin pregnancy, are debatable and have not been included in the guidelines as recognized risk factors for NH. Consequently, in this study, the impact of these factors on NH in neonates from mothers with GDM was explored.

It is well established that NH is an abnormal metabolic condition in neonates [14]. The capacity for glucose generation is limited in foetuses, and almost all foetal glucose is derived from the maternal supply [15–17]. Therefore, abnormal glucose metabolism in GDM women correlates with NH. In addition, dyslipidaemia is a critical characteristic of GDM [18–20]. Ryckman KK et al. reported that triglycerides (TGs) levels are greater in GDM women during pregnancy [21]. Therefore, exploring the link between maternal lipid metabolism and NH is crucial. Previous research has mainly focused on exploring the effects of maternal blood lipid indicators on SGA, LGA, macrosomia and other adverse pregnancy outcomes [22–24], and studies which have investigated the connection between maternal blood lipids and NH are in a minority [25, 26]. Consequently, this study included blood lipid indicators from GDM women in order to provide more evidence for the correlation between maternal blood lipids and the risk of NH in infants born to mothers with GDM. In addition, compared to conventional isolated indicators of blood lipids, more research now focuses on composite indicators. It has been reported that the total cholesterol (CHOL)/high density lipoprotein cholesterol (HDL-c) ratio is a more predictive risk indicator for cardiovascular disease compared to individual parameters [27]. Previous research has shown that an elevated apoprotein B/apoprotein A1 (Apo-B/Apo-A1) ratio was linked to a higher risk of cardiovascular diseases [28, 29]. Apo-B/Apo-A1 levels in early pregnancy significantly impact the occurrence of LGA [30]. However, it is unclear whether the Apo-B/Apo-A1 ratio can predict NH in infants born to mothers with GDM. Therefore, in addition to conventional isolated blood lipid indicators, this study included Apo-B/Apo-A1 and investigated its effect on NH in infants born to GDM women for the first time.

## Methods

### Study design and research populations

This study aimed to explore the influence of maternal blood lipids on NH in neonates born to mothers with GDM. A retrospective cohort study was conducted at the First Affiliated Hospital of Sun Yat-sen University (FAH-SYSU). Between January 2019 and February 2022, GDM women and their live neonates were recruited for the study. Participants meeting any of the following criteria were then excluded: [1] had a gestational age at delivery <35 weeks; [2] did not have detailed neonatal capillary blood glucose (CBG) records; or [3] lacked complete maternal blood lipid indicator records.

The present study received approval from the ethics committee of FAH-SYSU (Application ID [2022]451).

### CBG measurement and definition

Newborn CBG was defined as the glucose level in peripheral blood (neonatal heel blood) detected using a glucometer. CBG was measured routinely at birth and at 4, 8, and 12 h after delivery if any CBG was ≥3.0 mmol/L. Neonates with abnormal CBG conditions were monitored intensively as follows: [1] if blood glucose was < 3.0 mmol/L, CBG was checked every 30 min; [2] if blood glucose was ≤ 2.6 mmol/L, then subsequent treatments (breastfeeding, oral glucose or intravenous glucose) were initiated. Then, the CBG was remeasured 30 min after the intervention to check whether the hypoglycaemia of the newborn had resolved. Once the CBG of these neonates was ≥ 3 mmol/L, the CBG was measured every 4 h for a total of three measurements.

NH was defined as any CBG that was ≤ 2.6 mmol/L in the first 24 h after birth.

### Data collection

Maternal and neonatal information was recorded by doctors and midwives trained in the hospital’s medical records. The information was collected through the First Affiliated Hospital of Sun Yat-sen University (FAH-SYSU) digital information management system (HAITAI electronic medical records (EMRs) system). This EMRs system has the ability to export information on maternal demographic characteristics, clinical information, laboratory results and neonatal characteristics. Maternal demographic characteristics included age, pre-pregnancy body mass index (BMI), gestational weight gain and gestational age at delivery. Maternal clinical information included mode of delivery, twin pregnancy, multipara, assisted reproductive technology (ART) therapy, HDP, and thyroid disease. Moreover, data on insulin use during pregnancy were also collected. Maternal laboratory results for glucose and lipid profiles during the third trimester of pregnancy were compiled. For the neonates, the following characteristics were obtained: sex, appropriate for gestational age (AGA), SGA, LGA, foetal distress, neonatal asphyxia, and neonatal intensive care unit (NICU) admission.

The diagnostic criteria for GDM were based on the Standards of Medical Care in Diabetes of the American Diabetes Association (ADA) established in 2018 [1]. SGA was defined as birthweight < the 10th percentile, and LGA was defined as birthweight > the 90th percentile according to the semi-customized foetal growth curve based on the Chinese population for singleton neonates and the Chinese standard based on Jianping Chen et al.’s research for twin neonates [31, 32].

### Statistical analysis

Statistical analysis was conducted using SPSS 27.0 and R 4.1.0 software. Because of missing data for indices (pre-pregnancy BMI, gestational weight gain, oral glucose tolerance test (OGTT) fasting plasma glucose, OGTT 1-h post load plasma glucose, OGTT 2-h post load plasma glucose and HbA1c) in the original dataset, multiple imputation was used to generate a complete dataset based on existing indices, as reported previously [33]. Multiple imputation by chained equations was performed utilizing the mice package in R to provide 5 estimates of each missing value, generating 5 complete datasets. In the process of imputation, the random forest method was implemented, and the seed and action parameters were set to ensure data repeatability. The variables between the NH and control groups were compared using Student’s t test or the Mann‒Whitney U test for continuous variables and the χ^2^ test for categorical variables. Continuous variables are presented as the mean±standard deviation (SD) or median (interquartile range (IQR)), while categorical variables are presented as numbers (%). The composite lipid indices of the Apo-B/Apo-A1 ratio that deviated from a normal distribution were described as tertiles, and the data for this index were divided into three groups: < tertile 1, tertile 1-tertile 2, and > tertile 3, with P_33.33_ (0.491) and P_66.66_ (0.618) as the cut-off points. Statistically significant factors in univariate analysis were incorporated in a binary multivariate logistic regression analysis to calculate the odds ratio (OR) and 95% confidence intervals (CIs) to evaluate the risk of NH. Then, statistically significant factors in the logistic regression were used to build up a prediction model of NH. A nomogram was built for model visualization. The nomogram was assessed using the area under the receiver operating characteristic (ROC) curve (AUC). Statistical significance was defined as two-sided *P* values below 0.05.

## Results

**Fig. 1:**
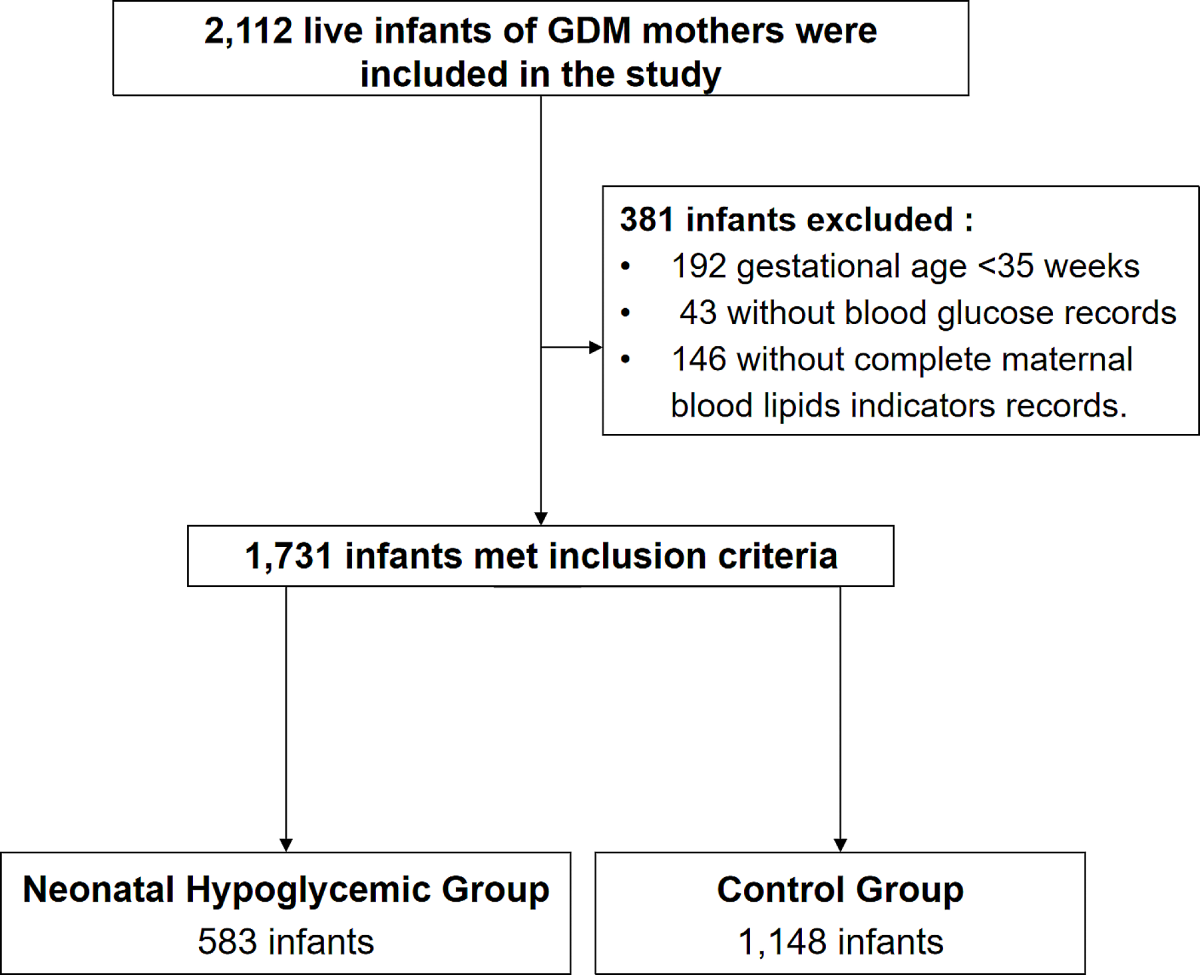
Process of clinical data collection and selection

A total of 2112 live neonates born to GDM mothers in the FAH-SYSU between January 2019 and February 2022 were included. According to the research protocol, 192 neonates who were delivered before 35 weeks and 43 neonates lacking available CBG records were excluded. 146 cases were excluded for missing complete maternal blood lipid records. Overall, 1731 infants were included in the statistical analyses, 583 of whom were in the neonatal hypoglycaemia (NH) group and 1148 of whom were in the control group (non-hypoglycaemic group) (Fig. 1).

### Maternal and neonatal characteristics

The characteristics for mothers and newborns of the NH and control groups were presented in Table 1. In terms of maternal demographic characteristics, mothers in the NH group were slightly older (34.59 ± 4.64 years vs. 33.44 ± 4.46 years, *P* < 0.001) and had slightly lower gestational age at delivery (37.95 ± 1.27 weeks vs. 38.24 ± 1.23 weeks, *P* < 0.001). Moreover, the proportion of multipara (52.0% vs. 45.9%, *P* = 0.019) was higher in the NH group. Maternal gestational weight gain and pre-pregnancy BMI were not different between the two groups.

For maternal clinical characteristics, the NH group had a greater proportion of twin pregnancy (13.2% vs. 5.0%, *P* < 0.001), ART therapy (27.4% vs. 19.1%, *P* < 0.001), HDP (9.6% vs. 5.7%, *P* = 0.004), and caesarean section (70.2% vs. 49.1%). No differences were found in thyroid disease and insulin use during pregnancy between the two groups.

Neonates in the NH group exhibited a greater proportion for SGA (11.0% vs. 8.9%) and LGA (8.2% vs. 5.1%), but a lower proportion of foetal distress (13.4% vs. 19.6%, *P* = 0.002). For other neonatal characteristics, there were no significant differences between the two groups.

**Table 1:** Characteristics of research populations

	Neonatal Hypoglycaemia (*n* = 583)	Control (*n* = 1148)	*P*
Maternal			
Age (years)	34.59 ± 4.64	33.44 ± 4.46	< 0.001^*^
Pre-pregnancy BMI (kg/m^2^)	21.86 ± 3.06	21.93 ± 3.06	0.656
Gestational weight gain (kg)	10.92 ± 4.36	10.96 ± 4.56	0.869
Multipara	303 (52.0)	527 (45.9)	0.019^*^
Gestational age at delivery (weeks)	37.95 ± 1.27	38.24 ± 1.23	< 0.001^*^
Twin pregnancy	77 (13.2)	57 (5.0)	< 0.001^*^
ART	160 (27.4)	219 (19.1)	< 0.001^*^
HDP	56 (9.6)	66 (5.7)	0.004^*^
Thyroid disease	30 (5.1)	65 (5.7)	0.738
Insulin use during pregnancy	3 (0.5)	18 (1.6)	0.097
Modes of delivery			< 0.001^*^
Vaginal delivery	174 (29.8)	584 (50.9)	
Caesarean section	409 (70.2)	564 (49.1)	
**Neonatal**			
Male	300 (51.5)	620 (54.0)	0.34
Birthweight for gestational age			0.011^*^
AGA	471 (80.8)	987 (86.0)	
SGA	64 (11.0)	102 (8.9)	
LGA	48 (8.2)	59 (5.1)	
Foetal distress	78 (13.4)	225 (19.6)	0.002^*^
NICU admission	110 (18.9)	236 (20.6)	0.443

The data are presented as the mean±SD, median (IQR) or n (%). Abbreviations: IQR, interquartile range; BMI, body mass index; ART, assisted reproductive technology; HDP, hypertensive disorders of pregnancy; AGA, appropriate for gestational age; SGA, small for gestational age; LGA, large for gestational age; NICU, neonatal intensive care unit * *P* value less than 0.05

### Maternal glucose and lipid metabolism profiles of the NH and control groups

The maternal metabolism profiles of glucose and lipids during pregnancy in the two groups were shown in Table 2. The NH group showed increased TGs level (3.58 ± 1.46 mmol/L vs. 3.43 ± 1.48 mmol/L, *P* = 0.048), apoprotein E (Apo-E) (58.63 ± 18.93 mg/L vs. 55.24 ± 16.14 mg/L, *P* < 0.001) and Apo-B/Apo-A1 ratio (0.58 ± 0.18 vs. 0.56 ± 0.16, *P =* 0.023) but lower level of HbA1c (5.18 ± 0.43 vs. 5.25 ± 0.45, *P =* 0.003) than the control group. Furthermore, newborns from the NH group had a decreased proportion of tertile 1 for Apo-B/Apo-A1 (33.1% vs. 33.45%) and tertile 2 (29.5% vs. 35.10%); however, a greater proportion of tertile 3 for Apo-B/Apo-A1 (37.4% vs. 31.45%). Other laboratory test results showed no significant differences between the two groups.

**Table 2:** Maternal metabolism profiles of glucose and lipid

	Neonatal Hypoglycaemia (*n* = 583)	Control (*n* = 1148)	*P*
OGTT fasting plasma glucose level (mmol/L)	4.59 ± 0.54	4.57 ± 0.50	0.547
OGTT 1-h post load plasma glucose level (mmol/L)	9.93 ± 1.24	9.95 ± 1.27	0.7
OGTT 2-h post load plasma glucose level (mmol/L)	8.80 ± 1.14	8.69 ± 1.18	0.061
HbA1c (%)	5.18 ± 0.43	5.25 ± 0.45	0.003^*^
HDL-c (mmol/L)	1.84 ± 0.38	1.86 ± 0.34	0.242
LDL-c (mmol/L)	3.73 ± 0.86	3.69 ± 0.80	0.378
CHOL (mmol/L)	6.55 ± 1.34	6.47 ± 1.23	0.237
TG (mmol/L)	3.58 ± 1.46	3.43 ± 1.48	0.048^*^
LP-a (mg/L)	124.00 (69.00-251.50)	120.00 (65.00-280.50)	0.948
Apo-A1 (g/L)	2.04 ± 0.34	2.06 ± 0.33	0.114
Apo-B (g/L)	1.16 ± 0.30	1.14 ± 0.27	0.098
Apo-E (mg/L)	58.63 ± 18.93	55.24 ± 16.14	< 0.001^*^
Apo-B/Apo-A1	0.58 ± 0.18	0.56 ± 0.16	0.023^*^
Apo-B/Apo-A1			0.021^*^
Tertile 1	193 (33.1)	384 (33.45)	
Tertile 2	172 (29.5)	403(35.10)	
Tertile 3	218 (37.4)	361 (31.45)	

The data are presented as the mean±SD, median (IQR) or n (%). Abbreviations: IQR, interquartile range; OGTT, oral glucose tolerance test; HbA1c, glycated haemoglobin; HDL-c, high density lipoprotein cholesterol; LDL-c, low density lipoprotein cholesterol; CHOL, total cholesterol; TGs, triglycerides; LP-a, lipoprotein (a); Apo-A1, apoprotein A1; Apo-B, apoprotein B; Apo-E, apoprotein E * *P* value less than 0.05

### Variation in neonatal glycaemia during the first 48 h

**Fig. 2:**
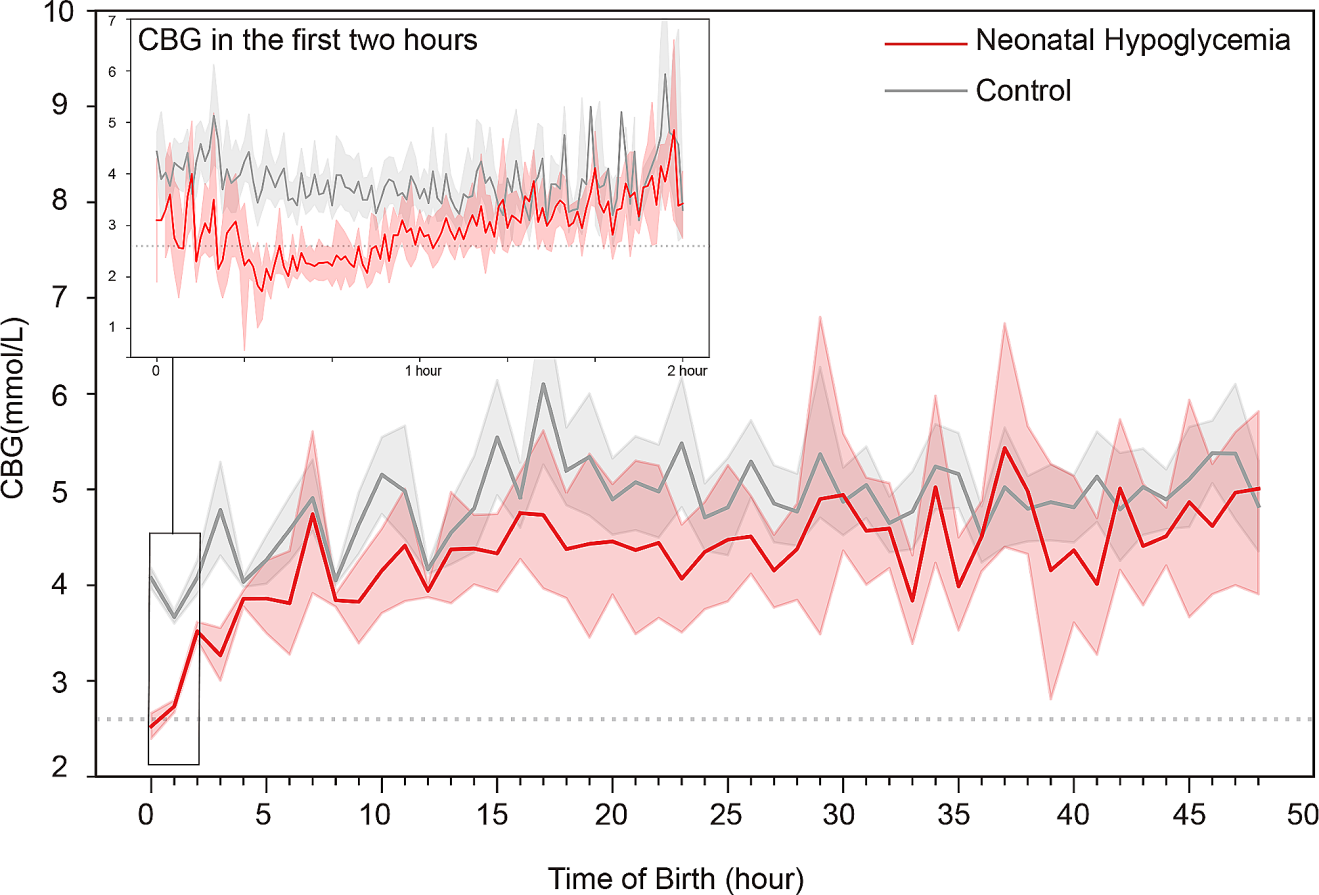
Glycaemic variation in neonates born after GDM in the first 48 h after birth. The middle solid line and shaded areas are the mean value and 95% CIs of the CBG in each hour, respectively. The inset shows the variation of CBG in each minute of the first two hours of life. The grey dotted line represents the glucose level of 2.6 mmol/L

To investigate the neonatal glycaemic concentration variation, the neonatal glucose levels of the two groups at all measuring points in the first 48 h after birth were compared. As shown in Fig. 2, the overall neonatal CBG level in the NH group was lower than that in the control group. In both groups, the CBG of neonates decreased rapidly in the first hour after birth and then increased gradually from the first to the second hour after birth. From the second hour on, the CBG of infants remained stable. In the NH group, 86.11% (502/583) of hypoglycaemia occurred within the first two hours after birth.

**Fig. 3:**
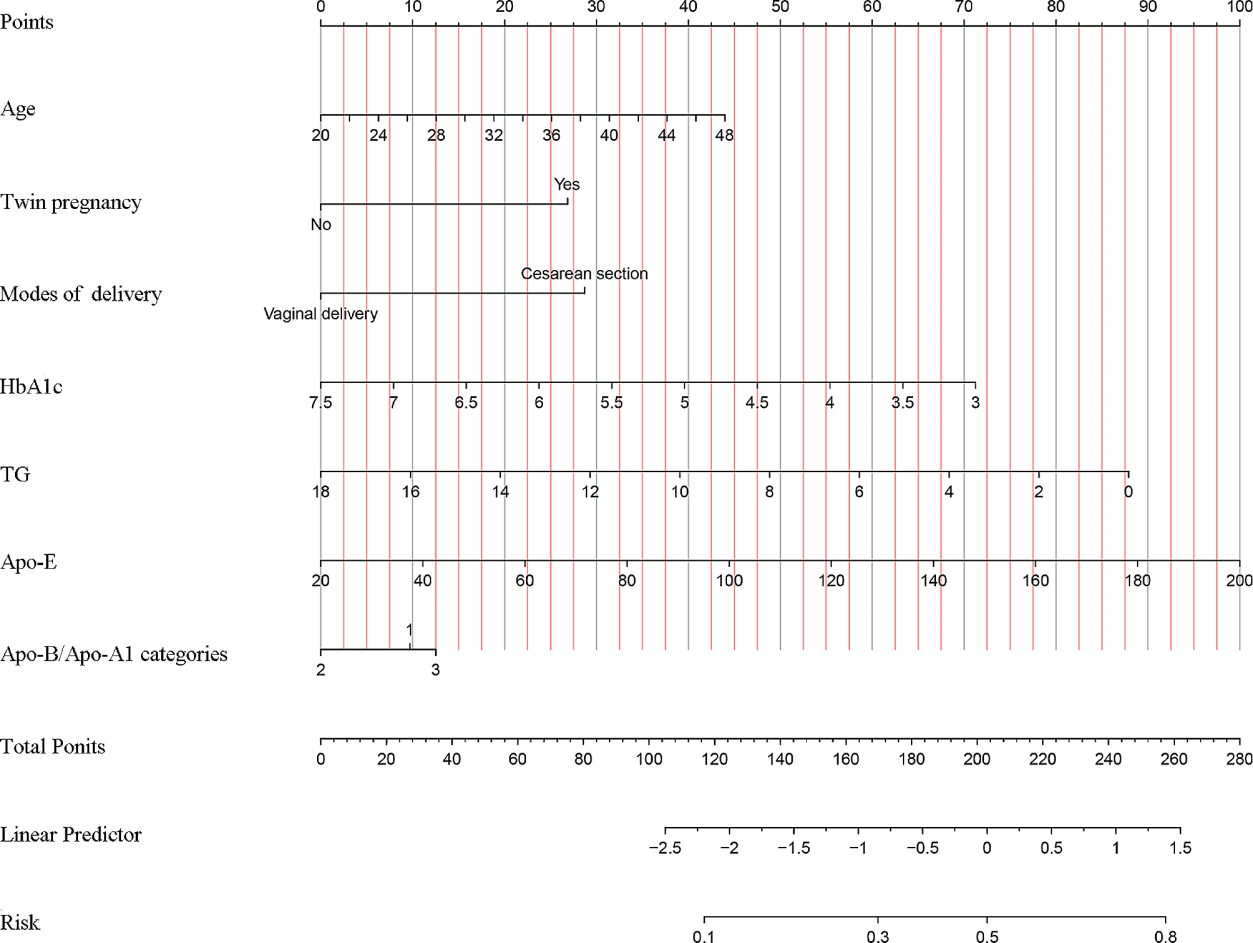
Nomogram predicting the risk of NH in newborns of GDM mothers. Seven indicators, including age, twin pregnancy, mode of delivery, HbA1c, TG, Apo-E and Apo-B/Apo-A1, were enrolled in the prediction model. The predictor points of each variable are projected to the top points scale and summed; then, the total points corresponding to the bottom risk scale represent the probability of NH for neonates born to GDM women

**Fig. 4:**
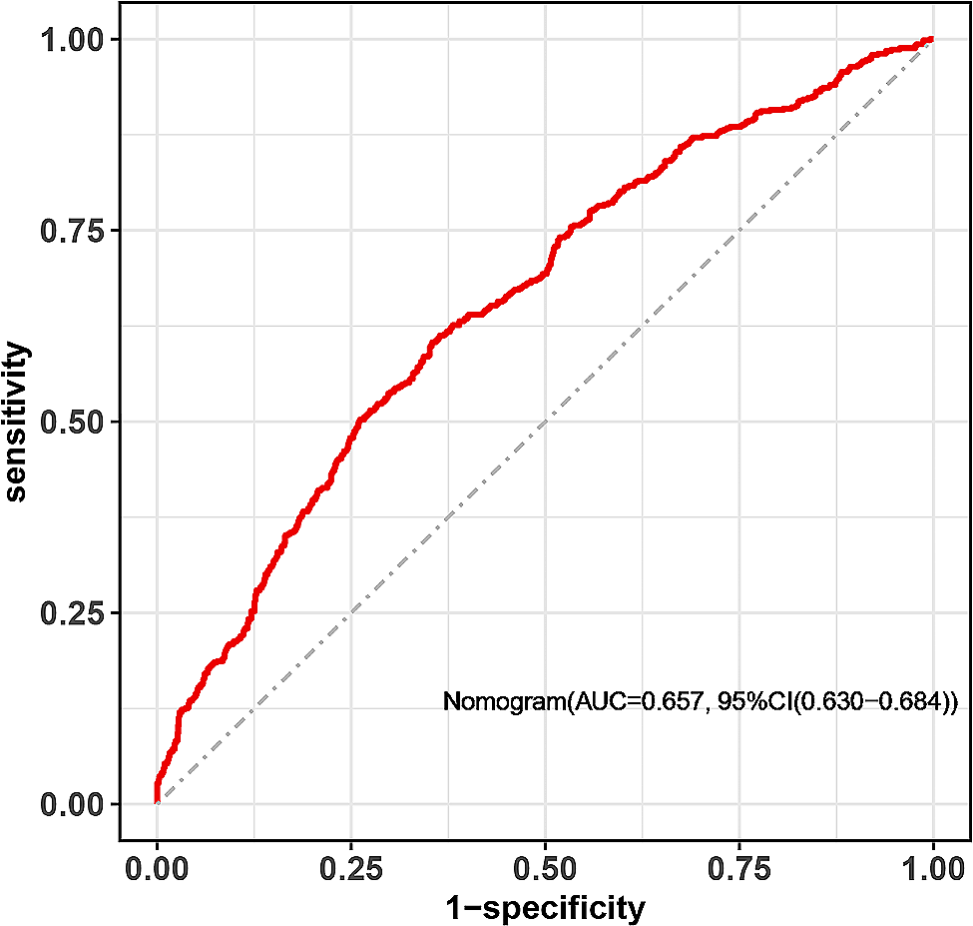
The AUC was calculated by ROC analysis to evaluate the discrimination ability of the nomogram. ROC, receiver operating characteristic; AUC, area under the ROC curve

### Predictive factors of NH in neonates born to mothers with GDM

A binary multivariate logistic regression analysis was further conducted to investigate the effects of factors for NH in infants from mothers with GDM (Table 3). The risk factors for NH included maternal age (OR: 1.03, 95% CIs: 1.003–1.058, *P =* 0.03), twin pregnancy (OR: 1.875, 95% CIs: 1.194–2.958, *P =* 0.007), caesarean delivery (OR: 2.042, 95% CIs: 1.617–2.585, *P*< 0.001), Apo-E (OR: 1.014, 95% CIs: 1.004–1.024, *P* = 0.004) and Apo-B/Apo-A1 tertile 3 (OR: 1.36, 95% CIs: 1.049–1.764, *P* = 0.02). However, higher maternal HbA1c (OR: 0.663, 95% CIs: 0.521–0.841, *P* = 0.001) and TGs (OR: 0.883, 95% CIs: 0.788–0.986, *P* = 0.028) levels reduced the risk of NH in infants born to mothers with GDM.

**Table 3:** Logistic regression analysis on the risk of NH in newborns of GDM mothers

	OR (95% CIs)	*P*
Age	1.03(1.003–1.058)	0.03^*^
Twin pregnancy	1.875(1.194–2.958)	0.007^*^
Caesarean delivery	2.042(1.617–2.585)	< 0.001^*^
HbA1c (%)	0.663(0.521–0.841)	0.001^*^
TG (mmol/L)	0.883(0.788–0.986)	0.028^*^
Apo-E (mg/L)	1.014(1.004–1.024)	0.004^*^
Apo-B/Apo-A1		
Tertile 2	Reference	
Tertile 1	1.276(0.984–1.656)	0.066
Tertile 3	1.36(1.049–1.764)	0.02^*^
Multipara	1.157(0.907–1.476)	0.242
Gestational age at delivery (weeks)	1.028(0.933–1.133)	0.585
ART	1.131(0.854–1.494)	0.387
HDP	1.457(0.978–2.164)	0.062
Birthweight for gestational age		
AGA	Reference	
SGA	1.185(0.824–1.692)	0.355
LGA	1.454(0.958–2.198)	0.076
Foetal distress	0.889(0.655–1.197)	0.442

Abbreviations: OR, odds ratio; CIs, confidence intervals; HbA1c, glycated haemoglobin; TGs, triglycerides; Apo-E, apoprotein E; Apo-A1, apoprotein A1; Apo-B, apoprotein B; ART, assisted reproductive technology; HDP, hypertensive disorders of pregnancy; AGA, appropriate for gestational age; SGA, small for gestational age; LGA, large for gestational age * *P* value less than 0.05

### Development of the predictive model for NH with a nomogram

According to the findings of the multivariable logistic regression analysis, seven variables including maternal Apo-B/Apo-A1, Apo-E, TG, HbA1c, age, twin pregnancy and caesarean delivery were independently associated with NH. These factors were subsequently incorporated into the predictive model for NH in pregnancy with GDM. A nomogram was constructed to visualize and assess the performance of the model (Fig. 3). The AUC was utilized to estimate the discriminatory ability of the nomogram; the AUC was 0.657 (95% CIs: 0.630–0.684) (Fig. 4).

## Discussion

The correlation between maternal blood lipids and hypoglycaemia in newborns of mothers with GDM was explored in the current study. Maternal Apo-B/Apo-A1 and Apo-E were identified as risk factors for NH. A nomogram containing maternal lipids, HbA1c, age, twin pregnancy and caesarean delivery was constructed to predict NH. In addition, the trajectory of glycaemia during the first 48 h in neonates born to GDM women was described, and NH was found to occur mainly within the first two hours after birth.

In recent studies, composite blood lipid indices have shown better predictive power for metabolic diseases than single blood lipid indices [34–36]. Deng, F. et al. proposed that the Apo-B/Apo-A1 ratio independently predicted erosion, plaque rupture, and thrombi in individuals with atherosclerotic cardiovascular disease [37]. Ying Zhao reported that the Apo-B/Apo-A1 ratio was a risk factor for metabolic dysfunction linked to fatty liver disease [38]. In addition, Zixuan Wang et al. reported that Apo-B/Apo-A1 levels during early pregnancy significantly influenced the development of LGA [30]. This study first investigates the relationship between Apo-B/Apo-A1 and NH in neonates of mothers with GDM.

Apo-A1 and Apo-B are positively correlated with high density lipoprotein (HDL) and low density lipoprotein (LDL), respectively, and can reflect the levels of HDL and LDL; thus, Apo-B/Apo-A1 has similar clinical significance to the LDL/HDL ratio. LDL carries cholesterol from the liver to the peripheral blood, while HDL transports extrahepatic cholesterol to the liver. Therefore, an elevated Apo-B/Apo-A1 ratio indicates an abnormal blood lipid profile, which leads to excessive deposition of cholesterol on the vascular wall to injure the vascular endothelium. In GDM mothers with high levels of Apo-B/Apo-A1, the utero-placental vascular endothelium may be damaged because of hyperlipemia, and this damage leads to an insufficient nutrient supply at the maternal–foetal interface [30], including a reduction in glucose transport to the foetus. Consequently, high levels of Apo-B/Apo-A1 increase the risk of NH in newborns from mothers with GDM. Compared with routine isolated blood lipid indices, Apo-B/Apo-A1 is a superior and easier-to-obtain lipid ratio parameter that is used to predict the risk of NH.

In this study, a higher TGs concentration was a protective factor against NH. TGs are hydrolysed into free fatty acids (FFAs) and glycerol by lipoprotein lipase (LPL), and FFAs are transported to the foetus through the placenta [39]. FFAs in the foetus enhance insulin resistance (IR), which promotes foetal glucose deposition. In addition, maternal FFAs can be converted into ketone bodies to reduce the consumption of glucose, which allows more glucose to be transported to the foetus. Moreover, glycerol from the TGs of mothers is used for the synthesis of glucose transported across the placenta to the foetus [39]. Therefore, a high level of maternal TGs leads to elevated neonatal glycaemia, which could decrease the incidence of NH.

The current study revealed that a high level of Apo-E increased the risk of NH. Apo-E is an apoprotein that plays a role in regulating lipid metabolism, and is mainly produced by the liver [40]. Therefore, Apo-E may affect the development of NH by influencing blood lipid levels. The Apo-E gene commonly has three alleles (ε2, ε3, and ε4), generating six genotypes (ε2ε2, ε3ε3, ε4ε4, ε2ε4, ε2ε3, and ε3ε4) [40]. The underlying mechanism by which Apo-E impacts neonatal glucose metabolism has not been fully clarified. A previous study discovered a negative correlation between Apo-E and blood glucose level, while a positive correlation with insulin level in GDM model mice [41]. Eline H van den Berg et al. reported that Apo-E promoted the liver to uptake TGs-rich lipoproteins and decreased TGs levels [40]. Thus, Apo-E may increase the risk of NH by downregulating TGs.

In the present study, infants from older mothers had a greater risk of hypoglycaemia than those born to younger mothers, confirmed by previous research [42]. The potential reason for this association is that placental efficiency may decrease in advanced-aged mothers [43]. Twin pregnancy was another risk factor for NH in the current study, which is also consistent with previous research [44, 45]. The underlying mechanism might the faster feto-maternal glucose consumption rate in twin pregnancy [46]. According to the analysis of data from this retrospective cohort, the caesarean delivery increased the risk of NH, which is consistent with prior study [12]. This may be due to the decrease in maternal and foetal blood glucose reserves resulting from preoperative fasting. HbA1c reflects the average level of glycaemia over the previous 8–12 weeks. In the current study, elevated HbA1c was correlated with a decreased risk of NH, which was in keeping with the findings of previous research [47]. However, Annie M. Dude et al. reported that NH was more likely to occur in newborns from mothers with higher HbA1c, but the HbA1c in that study was much higher than that of the present cohort [48]. Another study found that pregestational diabetic women with poor glycaemic control had increased NH risk [49], but their HbA1c level was also much higher than that in this study. Maternal glucose is transported through the placenta to the foetus. When maternal glycaemia increases, the foetus can obtain more glucose, which can increase the glucose reserve and reduce the risk of NH. These results indicated that appropriate maternal HbA1c level was a protective factor for NH.

The variation of glycaemia in neonates is important for health care providers to prevent NH. In the present study, the CBG of all neonates reached the lowest level within the first hour of birth, gradually increased after that, and then remained stable after the second hour of life. In the NH group, 86.11% (502/583) of hypoglycaemia occurred within the first two hours after birth. These findings are consistent with recent guidelines that transient low blood glucose levels often occur in the first 1–2 h after birth in nearly all infants of mammals [13]. Thus, intensive monitoring of CBG within two hours after birth is of great importance to prevent NH in neonates of mothers with GDM.

### Strengths and limitations

There are several strengths in the present study. Firstly, it was performed in a large cohort with well-preserved medical records and a broad range of clinical and laboratory characteristics in mother-neonate pairs. Based on the analysis of many cases, the results are credible. Secondly, previous research on the correlation between maternal blood lipid and NH has been insufficient. This research focused on maternal blood lipid profiles and revealed for the first time that Apo-B/Apo-A1 and Apo-E were risk factors for NH. This study provided new results and evidence for the clinical management of NH.

There are also several limitations. First, there was selection bias due to the research’s retrospective design. However, less than 5% of the data were missing for these variables, which minimizes bias in the analysis. Second, only maternal blood lipid levels during late pregnancy were obtained. The collection of blood lipids in the first and second trimesters to determine the trajectory of lipids during pregnancy might provide advanced predictive value for NH. Third, the present study was conducted in a single medical institution, and studies including pregnant women from multiple centres would contribute to strengthening the reliability of the results.

Elevated levels of blood lipid are common during pregnancy. The present study suggested that maternal Apo-B/Apo-A1, Apo-E, and TG were predictive factors for NH in infants from mothers with GDM and reminds clinicians to pay more attention to the blood lipid management of women with GDM to prevent NH. Moreover, a nomogram based on the study’s findings was established to assist clinicians build up a risk assessment table for NH. The trajectory of glycaemia for neonates born to GDM women indicated that intensive monitoring of CBG should be provided for these newborns within 2 h after birth.

This research revealed the clinical correlation between dyslipidaemia in GDM women and NH. Further studies should focus on the mechanism by which lipid parameters impact neonatal metabolism. In addition, future research including maternal blood lipid data during early and middle pregnancy would reveal lipid changes at different stages of pregnancy and the impact on the risk of NH. Multicentre research obtaining more convincing evidence will contribute to clinical guidelines for predicting and preventing NH.

## Conclusions

In conclusion, the present study revealed that maternal Apo-B/Apo-A1 and Apo-E levels were correlated with a higher risk of NH and reminds clinicians to improve the management of blood lipids in women with GDM during pregnancy. A nomogram consisting of maternal blood lipids, HbA1c, age, twin pregnancy and caesarean section was constructed, contributing to the clinical evaluation of the risk of NH in infants from mothers with GDM to prevent adverse outcomes of newborns. The description of neonatal glycaemia patterns suggested that intensive monitoring of CBG should be provided for infants born to mothers with GDM within 2 h after birth.

## Data Availability

The study’s data are included in the published article. The dataset analysed in this study can be obtained from the corresponding author upon request.

## Abbreviations

GDM: Gestational diabetes mellitus
NH: Neonatal hypoglycaemia
AGA: Appropriate for gestational age
SGA: Small for gestational age
LGA: Large for gestational age
TGs: Triglycerides
CHOL: Total cholesterol
HDL: c-High density lipoprotein cholesterol
LDL: c-Low density lipoprotein cholesterol
Apo: B-Apoprotein B
Apo: A1-Apoprotein A1
Apo: E-Apoprotein E
LP: a-Lipoprotein (a)
HbA1c: Glycated haemoglobin
OGTT: Oral glucose tolerance test
CBG: Capillary blood glucose
EMRs: Electronic medical records
BMI: Body mass index
ART: Assisted reproductive technology
HDP: Hypertensive disorders of pregnancy
NICU: Neonatal intensive care unit
FAH: SYSU-First Affiliated Hospital of Sun Yat-sen University
ADA: American Diabetes Association
SD: Standard deviation
IQR: Interquartile range
OR: Odds ratio
CIs: Confidence intervals
ROC: Receiver operating characteristic
AUC: Area under the receiver operating characteristic curve
HDL: High density lipoprotein
LDL: Low density lipoprotein
FFAs: Free fatty acids
LPL: Lipoprotein lipase
IR: Insulin resistance
